# Thyroid cancer incidences in the United Arab Emirates: a retrospective study on association with age and gender

**DOI:** 10.12688/f1000research.76121.1

**Published:** 2022-03-21

**Authors:** Asma Almansoori, Hauke Busch, Riyad Bendardaf, Rifat Hamoudi

**Affiliations:** 1 Sharjah Institute for Medical Research, University of Sharjah, Sharjah, 27272, United Arab Emirates; 2 College of Medicine, University of Sharjah, Sharjah, 27272, United Arab Emirates; 3 Luebeck Institute of Experimental Dermatology, University of Lüebeck, Lüebeck, 23562, Germany; 4 Oncology Unit, University Hospital Sharjah, Sharjah, 27272, United Arab Emirates; 5 Division of Surgery and Interventional Science, University College London, London, UK

**Keywords:** Thyroid carcinoma, Epidemiology, Cancer incidence; United Arab Emirates, Age Standardized Rate

## Abstract

**Background:** Thyroid cancer is the ninth most common malignancy worldwide, but the third most common malignancy in the United Arab Emirates (UAE) *.* To our knowledge, this is the first UAE nationwide study aimed at presenting incidence rates of thyroid cancer at the national level of UAE based upon data from the national cancer registry and GLOBOCAN.

**Methods:** Between 2011 and 2017, a total of 2036 thyroid cancer cases from UAE patients were registered, of which 75.3% were female and 24.7% male patients.

**Results: **The results showed 6.6% increase in thyroid cancer cases in the UAE from 2011 to 2017 (p < 0.001) with a rise of approximately 400 cases per year from 2011 to 2040. Age standardized rate calculations showed increase in prevalence from 1.18 in 2011 to 4.32 in 2017 but decreases in incidence from 1.05 in 2011 to 0.15 in 2017. This trend is confirmed by the predictive model showing increase in incidence from 0.15 in 2017 to 0.64 by 2040. Gender was shown to be significantly associated with thyroid cancer. The female to male ratio was significantly higher in Emirati patients (4.86:1) (p < 0.001) than expat patients (2.47:1) (p < 0.01). Interestingly, expat patients contributed to the majority of thyroid cancer cases despite having lower female to male ratio. The age at diagnosis was significantly associated with thyroid cancer (p = 0.03) with the highest frequency diagnosed at 35-39 years of age. Globally, data from the predictive model showed that Asia had the highest rate of increase per year and UAE the lowest.

**Conclusions: **The slight increase in thyroid cancer prevalence and incidence, together with the different female to male ratio and diagnosis at younger age warrants further investigation at the molecular level from UAE thyroid cancer patients to elucidate the molecular basis of thyroid cancer.

## Introduction

Over the last decades, the overall worldwide incidence of thyroid cancer has increased significantly. ^1^ In the United States, thyroid carcinoma has become the most rapidly increasing cancer amongst both genders. The malignancy has been on the rise by an average of 1.9% over the ten last years with rising mortality rate of 0.7% annually between 2007-2016. According to the National Cancer Institute statistics, it constitutes 3.0% of all cancer types and the newly diagnosed cases were 15.8 per 100,000 among both genders per year. ^2^ In 2016, there was an estimate of 822,242 persons living with thyroid carcinoma in the United States. Ultrasound is most commonly used in detecting papillary thyroid cancer, however the diagnosis of papillary thyroid carcinoma should not only use ultrasound as it is not accurate predictor of papillary thyroid carcinoma and should be complimented with other diagnostic tests such as fine needle aspirate. The reasons for the increase in thyroid cancer are unclear and have been attributed to improved diagnosis of papillary thyroid cancer through the use of fine needle aspirate. It was also estimated that 52,070 new cases will be diagnosed in 2019 and approximately 2,170 deaths among both genders in the USA. ^3^ The highest incidence rate was between 45-54 years of age with a median age of 51 at the time of diagnosis. Patients aged between (75-84), have the highest mortality rate of 27.4% with a median age of 73 at death. ^3^ In the UAE, the incidence of thyroid cancer has increased at a rate that exceeds the incidence in neighboring countries and Western populations. ^4^

Thyroid cancer is the top ranked endocrine malignancy and the second most common female malignancy in the United Arab Emirates. ^5^ According to the Ministry of Health and Prevention (MOHAP), thyroid cancer is among the top ranked cancer cases in both genders besides Breast and Colorectal cancer. Between 2011 and 2017, thyroid cancer incidence has an overall peak from 4.37% to 9.99% among all new cancer cases. ^6^

According to the Global Cancer Observatory (GLOBOCAN) ^7^ collected in 2018, a total of 360 thyroid cancer cases were diagnosed in the UAE, 279 (77.5%) among females and 81 (22.5%) among males. The age-standardized incidence rate (ASR) per gender was 13.6/100,000 in females and 1.9/100,000 in males 17-20. ^7^ In 2020, a total of 405 (8.4%) cases were reported. The age-standardized incidence rate (ASR) per gender was 12.5/100,000 in females and 2.0/100,000 in males. ^7^

At the regional level in Gulf Cooperation Council Countries (GCC), ^8^ thyroid carcinoma is the fifth most common malignancy in both genders and again the second most common malignancy in females. During 1998–2007, a total of 5587 (5.9%) were recorded with an overall age-standardized incidence rate (ASR) of 5.9/100,000 in females and 1.8/100,000 in males. Thyroid cancer incidence in GCC was projected to continue increasing by 24% in males and 63% in females over the 10 years period. ^5^^,^^7^^,^^9^ Thus, investigating existing estimates for thyroid cancer in the UAE and worldwide is useful for future studies of the disease.

With the absence of recent national studies of UAE cancer epidemiology, and in compliance with the World’s Health Organization recommendations to establish cancer baselines and monitoring trends to address tumor related to thyroid gland, ^10^ therefore, this study’s main aim is to compile and analyze the estimates of thyroid malignancies across the UAE and compares them to worldwide incidence of thyroid cancer based on multiple attributes including ethnicity, gender and age groups.

## Methods

### Data collection

Global data was collected using the GLOBOCAN repository. ^7^ Estimates of global future cancer incidence was carried out using tools available from the Cancer Tomorrow website, which is part of the Global Caner Observatory software suite. ^11^^,^^12^

Local UAE data in this study were collected from the UAE National Cancer Registry (UAE-NCR) between 2011-2017. The inclusion criterion is for primary thyroid carcinoma. The UAE-NCR is systematically collecting, storing, summarizing and analyzing cancer estimates of UAE populations on annual basis. It provides population-based incidence rates in accordance to demographics, cancer staging and treatment information and in compliance with international coding and registration protocols (ICD-10 and ICD-0). The UAE-NCR’s abstractors used active and passive data collection methods. The active method involves data abstraction through regular visits to MOHAP departments. The passive method involves notifications by healthcare providers on submitted forms containing patient’s data obtained from: Health information management system (HIM), pathology reports, Abu Dhabi Health Authority (HAAD) central cancer registry, Dubai Health Authority (DHA) central cancer registry. Crude incidence and crude mortality rates were computed and expressed as an annual mean per 100,000 residents; using the total UAE population estimated by the Department of Economic and Affairs, population division of the United Nations. ^13^ In addition, predictive model for total malignancies in the UAE was constructed by applying linear regression to UAE data collected from 2011 to 2017 obtained from the (UAE-NCR). In order to determine the rate of change of thyroid cancer per year, gradient analysis derived from the linear regression equation was applied to the data derived from the model. For the ASR calculations, we used population data obtained from US Census Bureau and UAE Federal Competitiveness and Statistics Centre.

### Statistical analysis

In this study, categorical variables were reported as count and percentages. Continuous variables were presented as mean ± standard deviation. Group comparison was carried out using Student t-test. Correlation was carried out using Pearson and Rank correlations where appropriate. Unsupervised hierarchical clustering was carried out using Euclidean distance measure and average linkage. Statistical analysis was carried out using a mixture of R and SPSS. P < 0.05 was taken to be statistically significant.

## Results

### Global and UAE thyroid cancer demographics

**Figure 1.**
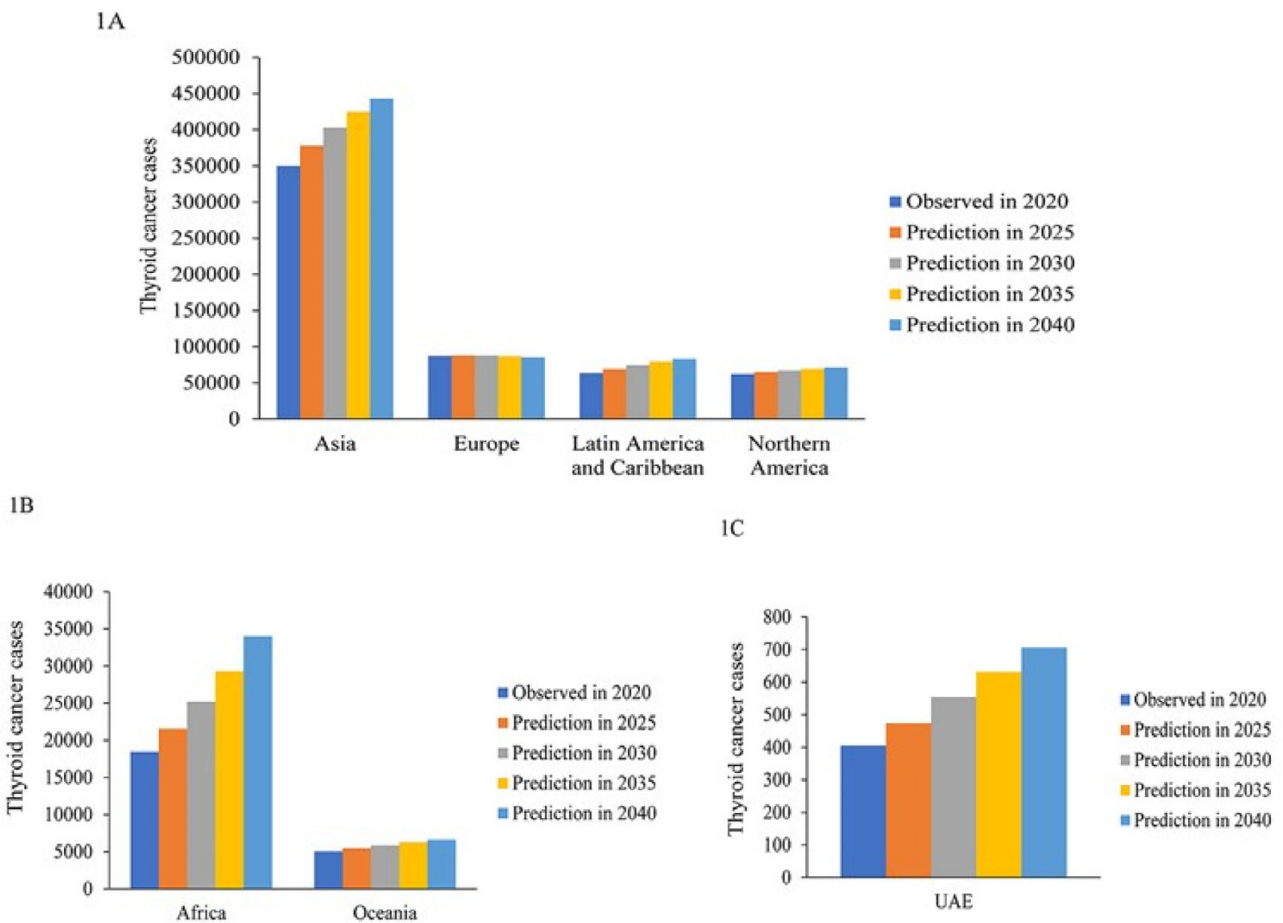
Global thyroid cancer demographics using the predictive model based on GLOBOCAN data from 2020 to 2040.

The data from Figure 1 show that the number of thyroid cancer cases rises globally, with the exception of Europe, at a steady rate from 2020 to 2040. The largest number of thyroid cancer cases is from the Asian continent as shown in Figure 1A. Figure 1C show that the UAE follows the global trend of steady but small increase in thyroid cancer cases.

**Figure 2.**
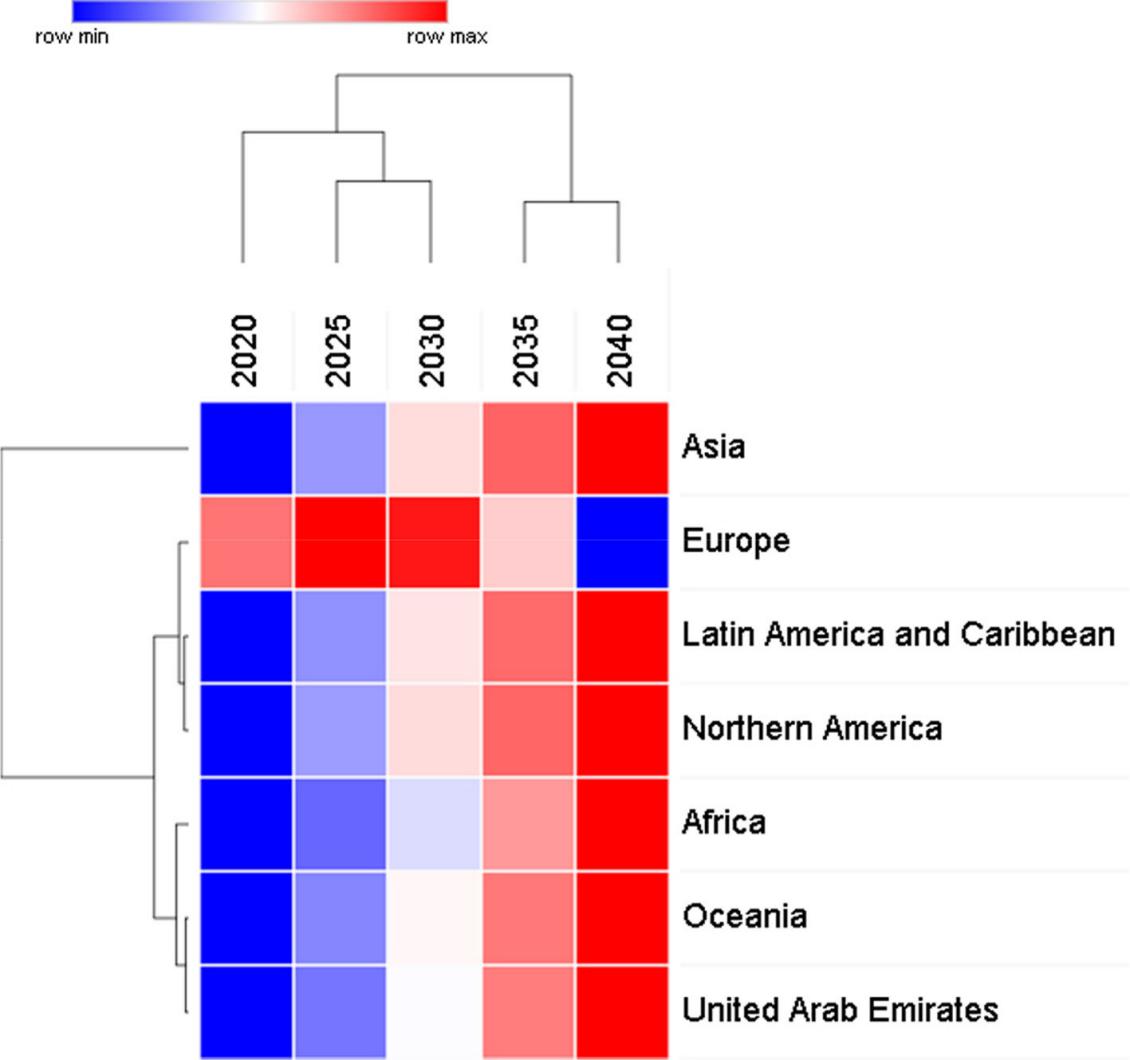
Two-way unsupervised hierarchical clustering of predicted data for global thyroid cancer incidence from 2020 to 2040.

Table 1 data is generated using linear regression models for each population and calculating the rate of change of thyroid cancer cases per year from the gradient of the linear regression equation for each population. The data indicates that overall, the rate of increase of thyroid cancer cases is small compared to other cancers. The unsupervised hierarchical clustering in Figure 2 is based on the predictive model and shows that there is a steady increase in the number of thyroid cancer cases in all regions except in Europe where the cases increase at a faster rate than the other region until 2030 but then from 2030 to 2040 the cases will start to decrease. 

**Table 1.** The rate of change of thyroid cancer incidence from 2020 to 2040 using predictive model data.

Region	Observed in 2020	Prediction in 2025	Prediction in 2030	Prediction in 2035	Prediction in 2040	Rate of change of the number of thyroid cancer cases per year (2020-2040)
Asia	349897	377585	402580	424769	442929	4665
Africa	18457	21562	25173	29318	34006	3885
Latin America	63368	69064	74411	79219	83361	1002.8
North America	62256	64960	67260	69292	71055	438.6
Oceania	5062	5474	5875	6262	6635	78.7
UAE	405	474	554	632	706	15.2
Europe	87162	87656	87557	86785	85490	-84.3

### Global and UAE thyroid cancer gender analysis

**Figure 3.**
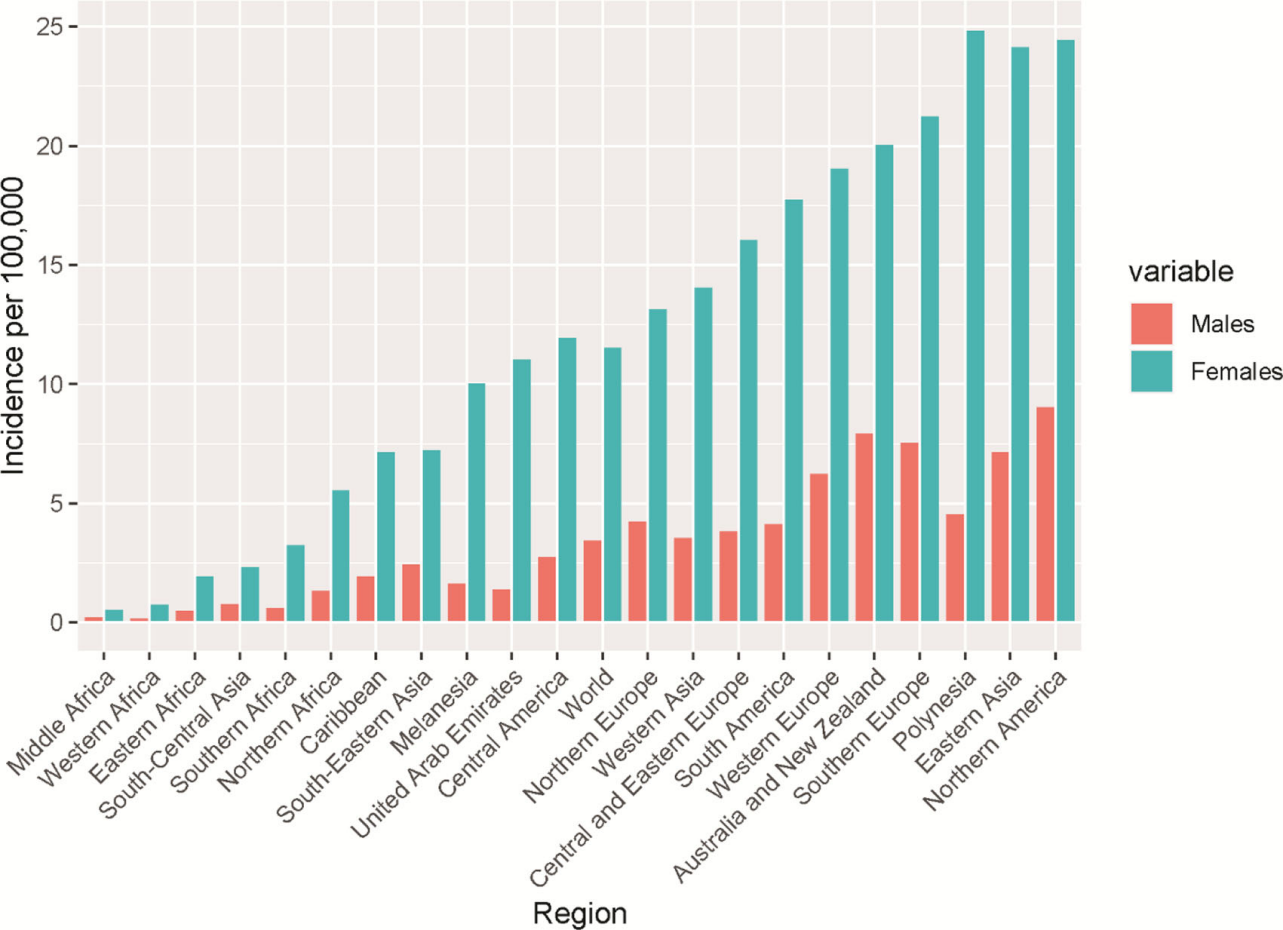
Number of male and female thyroid cancer incidence for selected world regions based on the Global Cancer Observatory data for 2020.

Figure 3 shows that thyroid cancer has a high ratio of female to male globally across various continents suggesting that gender might be a risk factor globally. Interestingly, the highest thyroid cancer incidence was found in Polynesia with the lowest in Africa. The average world-wide incidence of thyroid cancer is 11.6 and 3.5 for females and males, indicating an approximately 3 times higher incidence rate in female.

Taken together, Table 1 confirms the data in Figures 2 and 3 in that the largest increase of thyroid cancer cases is in Asia followed by Africa and Latin America. UAE shows the smallest rate of change at 15.2 and Europe has a negative rate of change due to the predicted decrease in cases from 2030 to 2040.

### Predicted total malignancy incidence in the UAE from 2011 to 2040

In order to predict the total malignancies, linear regression model was used to calculate all cancer incidence rates from 2011 to 2017. The data from the linear regression model was compared with that from the predicted model as shown in Table 2.

**Table 2.** Actual and predicted cancer incidence in UAE.

Year	Predicted total malignancies	Actual total malignancies	Thyroid cases	Percentage of predicted thyroid cancer (%)
2011	2688	2284	98	3.65
2012	2954	3094	188	6.36
2013	3221	3574	282	8.76
2014	3221	3610	314	9.75
2015	3487	3744	344	9.87
2016	3754	3982	398	10.60
2017	4020	4123	412	10.25
2020 ^*^	4820	-	405	8.40
2025 ^*^	6153	-	474	7.70
2030 ^*^	7485	-	554	7.40
2035 ^*^	8818	-	632	7.17
2040 ^*^	10151	-	706	6.95

* Represents predicted data.

Table 2 shows that the linear regression model prediction matches the actual data as seen by the significant correlation between predicted and actual data between 2011 and 2017 (r = 92.5, p = 0.003). In addition, Table 2 shows an increase of 6.6% between 2011 and 2017 which is similar to that in Table 1, however it drops to 3.3% of total thyroid cancer in UAE between 2011 and 2040, which is a slight increase.

### Thyroid cancer incidence and prevalence in UAE from 2011 to 2040

Table 3 shows slight but steady increase in the incidence and prevalence in UAE from 2011 to 2040 with the average increase for prevalence is 4.02 and incidence is 0.52.

**Table 3.** Prevalence and incidence cases of thyroid cancer per 100,000 in the UAE.

Year	Prevalence per 100,000	Incidence per 100,000
2011	1.18	ND
2012	2.18	1.05
2013	3.16	1.05
2014	3.42	0.35
2015	3.7	0.32
2016	4.22	0.57
2017	4.32	0.15
2020 ^*^	-	ND
2025 ^*^	-	0.68
2030 ^*^	-	0.76
2035 ^*^	-	0.71
2040 ^*^	-	0.64

In this study, the prevalence of thyroid cancer is taken to be the proportion of patients within the UAE population that have thyroid cancer at a particular time and incidence is taken to be the number of past, current and future cases of thyroid cancer.

* Represents predicted data.

Table 4 shows that between 2011 and 2017, a total of 2036 newly diagnosed thyroid cancer cases were recorded by UAE-NCR. Out of those (n = 634, 31%) are Emirati and (n = 1402, 69%) are expats.

**Table 4.** UAE thyroid cancer percentage compared to total malignancies from 2011 to 2017.

Year	Total malignancies	Thyroid cancer cases	Emirati patients	Expat patients
2011	2284	98	39	59
2012	3094	188	63	125
2013	3574	282	87	195
2014	3610	314	88	226
2015	3744	344	110	234
2016	3982	398	124	274
2017	4123	412	123	289
Total	24411	2036	634	1402

Table 5 shows that thyroid cancer patients between 2011 and 2017 comprised of 75.2% females and 24.7% males. Age standardized rate calculations showed slight increase in prevalence from 1.18 in 2011 to 4.32 in 2017 but a decrease in incidence from 1.05 in 2012 to 0.15 in 2017. This trend is confirmed by the predictive model showing the incidence to be 0.64 by 2040.

**Table 5.** Thyroid cancer distribution by nationality in the UAE from 2011 to 2017.

Gender	Frequency	Percentage
Male	504	24.7
Female	1532	75.2
Total	2036	100

### Percentage change of thyroid cancer within UAE

**Figure 4.**
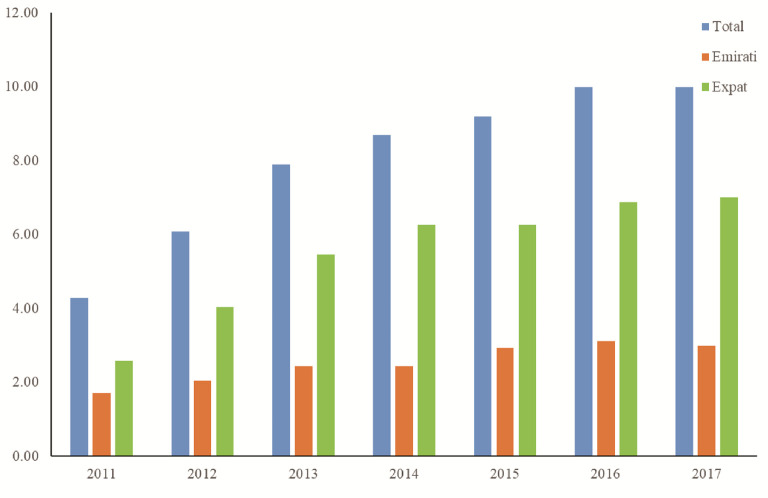
Percentage thyroid cancer demographics within the UAE population.

Figure 4 show the rise of thyroid cancer is in line with the rise of total malignancies within the UAE with the percentage rise of 5.7% for total cases comprising of 1.3% for Emirati and 4.4% for Expat patients between 2011 and 2017. In addition, Figure 4 show steady rise in both Emirati and expats patients with expats providing more of the contribution to the total number of cases of thyroid cancer.

**Figure 5.**
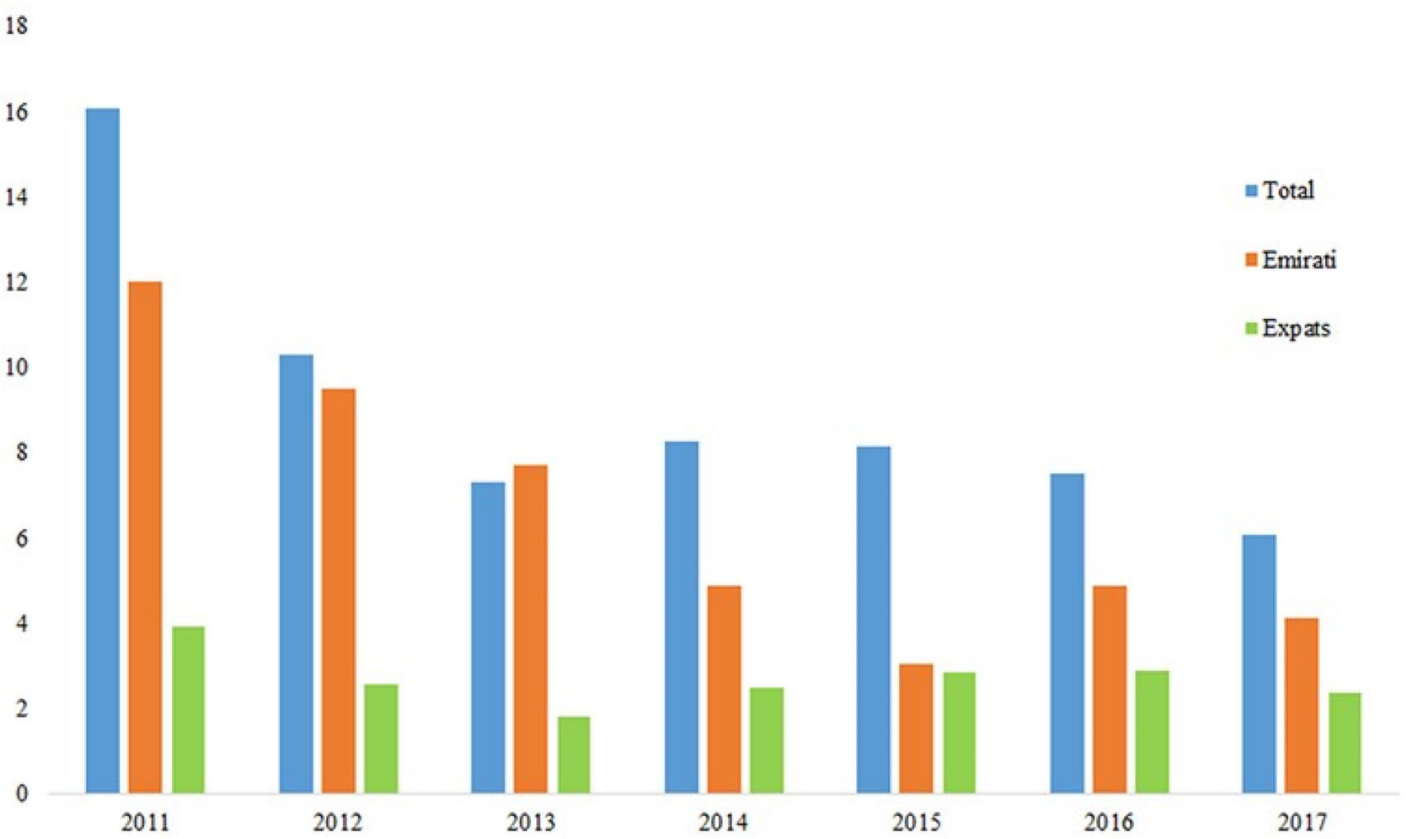
Female to male thyroid cancer incidence ratio per nationality within the UAE.

Figure 5 shows that the female to male ratio in 2011 was high but leveled from 2012 to 2017 with the Emirati ratio being highest than from expats throughout that period. The trend in Figure 5 is confirmed by data shown in Table 6, which indicates that overall, thyroid cancer incidences are higher in females than males. This is shown to be the case from 2011 to 2017 with 2011 having the highest ratio of 12 for Emirati and 3.92 for expat patients. Generally, the Emirati patients have higher ratio than expats. Using independent Student’s t-test, the female to male ratio is shown to be significant for both Emirati (p < 0.0001) and expats (p < 0.001), respectively.

**Table 6.** UAE gender distribution of thyroid cancer incidence from 2011 to 2017.

Year	Emiratis				Expats			
	Total	Males	Females	Female: Male ratio	Total	Males	Females	Female: Male ratio
2011	39	3	36	12.00	59	12	47	3.92
2012	63	6	57	9.50	125	35	90	2.57
2013	87	10	77	7.70	195	69	126	1.83
2014	88	15	73	4.87	226	65	161	2.48
2015	110	27	83	3.07	234	61	173	2.84
2016	124	21	103	4.90	274	70	204	2.91
2017	123	24	99	4.13	289	86	203	2.36

Taken together, the data show that in all populations thyroid cancer have high female to male ratio, however within the UAE the Emirati patients have higher ratio than the expats group, but the expats contribute more towards the thyroid cancer cases within the UAE.

### Age-group distribution in UAE

Table 7 and the cluster in Figure 6A show that in UAE the peak thyroid cancer diagnosis tends to be within the young age group of 35-39 (p = 0.03) with 17.5% of all thyroid cancer patients diagnosed between 2011 and 2017, followed by the 30-34 age group comprising of 15.9% of all thyroid cancer cases. Figure 6B confirms the findings with the highest number of patients of thyroid cancer are diagnosed with the age of 30-34 and 35-39. Also, Table 7 and Figure 6B show that the age range 30-44 account for 47.8% of all thyroid cancer patients.

**Table 7.** Overall age-specified distribution of thyroid cancer in UAE from 2011 to 2017.

Age group	Number of thyroid cases
1-4	3
5-9	2
10-14	11
15-19	29
20-24	87
25-29	201
30-34	323
35-39	355
40-44	293
45-49	227
50-54	183
55-59	131
60-64	80
65-69	46
70-74	31
75-79	13
80-84	9
85-89	4
90-94	3
115-119	1
Unknown	4

**Figure 6A.**
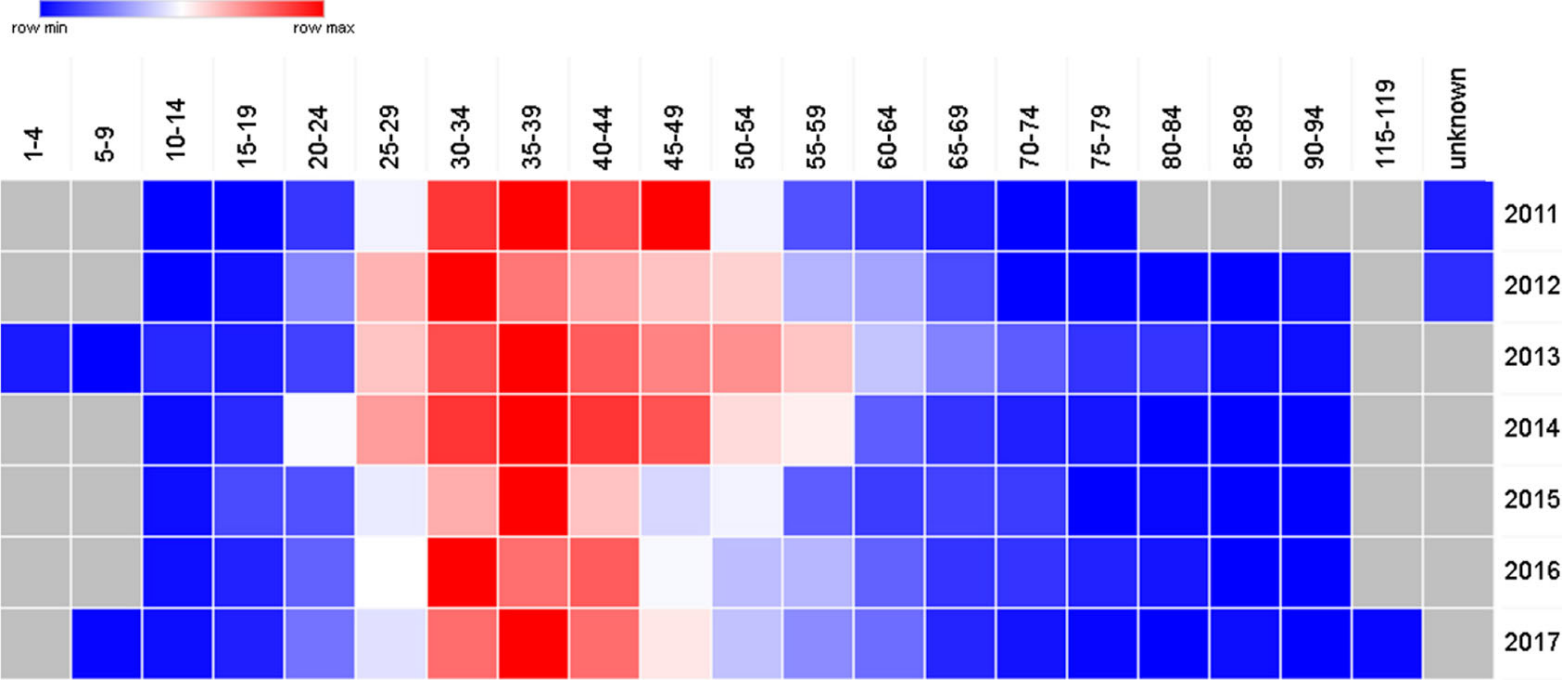
Heatmap representation for the age distribution of thyroid cancer in UAE from 2011 to 2017.

**Figure 6B.**
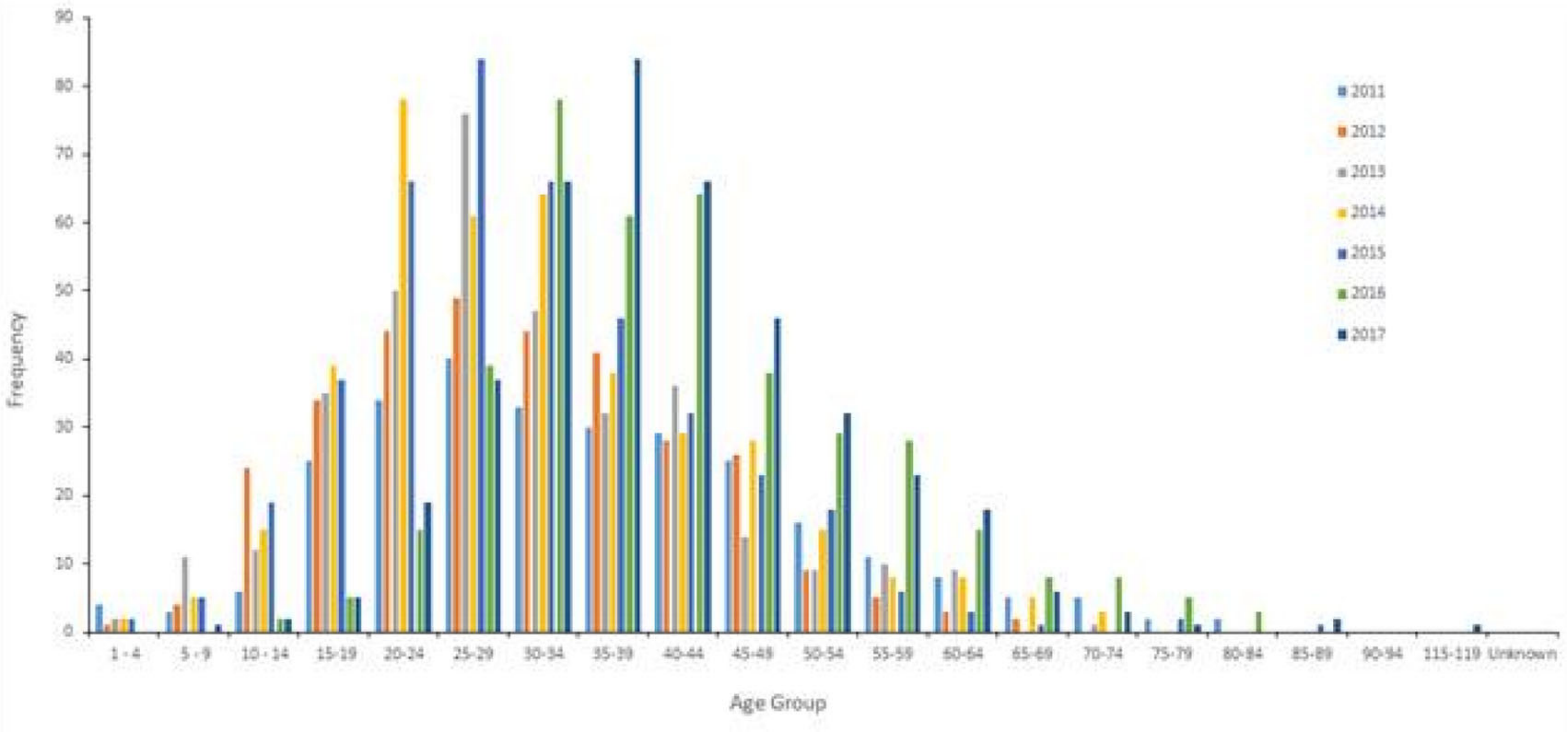
Graphical representation of the age distribution of thyroid cancer in UAE from 2011 to 2017.

## Discussion

To our knowledge, this is the first study focused on thyroid cancer incidence in the UAE. The data showed that thyroid cancer constitutes the highest percentage of endocrine malignancies. ^13^ The incidence rate of thyroid cancer has increased from 4.37% in 2011 to 9.99% in 2017, indicating a slow but steady increase compared to worldwide incidence. This is supported by the recent article showing incidence of thyroid cancer in the UAE between 2011 and 2017 showed an increase. ^13^

The results showed that both globally and locally within the UAE, thyroid cancer is associated with gender, in that the disease occurs in more female patients. Since thyroid cancer is an endocrine disease, the higher female incidence might be partly attributed to female hormone fluctuations as it was shown that some of the female hormones such as estrogen are associated with thyroid cancer. ^14^ The higher incidence in UAE females ^5^ may be associated with a mixture of endocrine genetic factors together with other environmental factors such as obesity, which is prevalent in UAE, smoking and a sedentary lifestyle. ^15^^,^^16^

This increase in incidence of thyroid cancer in the UAE female population is the main reason why the results showed that although thyroid cancer incidence in UAE is low, the prevalence in the UAE female population between 2011 and 2017 is higher than the rest of the world.

Interestingly, the data showed that female to male ratio is generally high in Emirati than expats, but with expats contributing to more cases of thyroid cancer. Also, the data show that male thyroid cancer incidence is low, leading to high female to male incidence ratio with the Emirati having higher ratio than expats. However, the ratios stabilizing after 2012, probably due to the UAE adopting more sensitive thyroid cancer diagnostic testing including genetic testing. ^17^^,^^18^ The differences between Emirati and expats ratio may be due to a mixture of the population pyramid of expats arriving to the UAE for economic purposes and possibly medical tourism. ^19^

In addition, the analysis showed that another risk factor for thyroid cancer in the UAE is age, where the highest frequency (17.5%) of thyroid cancer patients tends to be between 35-39 years of age and around 47.8% of the patients tend to be between 30-44 years of age. This might be explained by the fact that the younger generation in UAE adopted changes in lifestyle, including poor diet, which may be deficient in iodine, ^8^ however further studies are needed to confirm such association. Taken together, the results showed that the peak incidence of thyroid cancer was during approximately the third and early fourth decades of life amongst the UAE patients, which is a decade behind neighboring Arabian Gulf countries, ^8^ where thyroid cancer patients are diagnosed in their fourth decade of life. Taken together, the strengths of the study included data related to the thyroid cancer cases diagnosed in UAE from 2011 to 2017 and made predictive observations for the years 2020 to 2050. In addition, this is the first of the kind to report the thyroid cancer incidence in UAE population. The major limitations include the lack of availability of patient characteristics such as BMI, family history as well as other clinical characteristics. Overall, the incidence of thyroid cancer in UAE showed a small but steady increase, probably due to better diagnostic tests, as the data showed that the rate of increase is similar to other malignancies in the UAE. In addition, the methodologies used in this study may be applied to investigate other chronic complex diseases in the UAE from data stored in disparate sources.

In conclusion, this study showed that the incidence of thyroid cancer in the UAE increases at a slow but steady rate per year in line with worldwide data. The study identified that gender is associated with increase in risk of thyroid cancer probably due to a combination of genetic and environmental factors. The study also showed that although the female to male ratio of thyroid cancer is higher in Emirati, the contribution of the majority of thyroid cancer cases in UAE come from the expats. In addition, the study showed that more than a third of UAE thyroid cancer patients are diagnosed below the age of 40. In addition, the predictive models used in the study showed that future trend of thyroid cancer incidence in Europe may be different than the rest of the rest of the world.

## Data availability

### Underlying data

All data underlying the results are available as part of the article and no additional source data are required.

### Extended data

Figshare: Thyroid cancer incidences in the UAE

https://doi.org/10.6084/m9.figshare.19232841.v1 ^20^

This project contains the following extended data:

• Supplementary data for figures.xlsx

Data are available under the terms of the Creative Commons Zero “No rights reserved” data waiver (CC0 1.0 Public domain dedication).

## Competing interests

No competing interests were disclosed.

## Grant information

The authors declared that no grants were involved in supporting this work.
