# Supplementary material for: Thyroid cancer incidence in the United Arab Emirates: a retrospective study on association with age and gender
Source: F1000Res. 2022 Apr 11;11:338. Originally published 2022 Mar 21. [Version 2] doi: 10.12688/f1000research.76121.2 (PMC9065926; doi:10.12688/f1000research.76121.2)
Supplement: Peer review history

# Peer review: Reviewer response for version 2

**DOI:** 10.5256/f1000research.126337.r134593

**Published:** 2022-05-03

**Authors:** Jan Bures

**Affiliations:** 1 2nd Department of Internal Medicine-Gastroenterology, Faculty of Medicine in Hradec Kralove and University Hospital, Charles University, Hradec Kralove, Czech Republic

Thank you for the opportunity to review this interesting important paper.

 I have no major comments.

**Minor comments:**

- Was there any significant difference in the prevalence of thyroid cancer between Emirates (Abu Dhabi, Sharjah, Dubai, Ajman, Fujairah, Ras Al Khaimah, Umm Al Quwain)?
- Is there the same availability of health care service for Emirati and expat patients? Is there any (active) screening programme for thyroid cancer in UAE? Could you exclude selection bias?
- Please, provide rates (percentage) of the particular types of thyroid cancer (papillary, follicular, medullary, anaplastic… ), including female / male ratio.
- Authors hypothesized that high female / male ratio could be explained by female hormone fluctuations. Please, provide separately gender ratio in subgroups "younger than 54 years" and "55 years and older" to support this hypothesis.
- Table 5 deals with "gender" not "nationality".
- Is kitchen salt (NaCl) artificially supplemented with iodine in UAE? If yes, when did this approach start? Please, provide this information.

**Conclusion**

 I suggest Approving this paper for indexing after minor revision.

Is the work clearly and accurately presented and does it cite the current literature?

Yes

If applicable, is the statistical analysis and its interpretation appropriate?

Yes

Are all the source data underlying the results available to ensure full reproducibility?

Yes

Is the study design appropriate and is the work technically sound?

Yes

Are the conclusions drawn adequately supported by the results?

Yes

Are sufficient details of methods and analysis provided to allow replication by others?

Yes

Reviewer Expertise:

Internal Medicine, Gastroenterology, Oncology

I confirm that I have read this submission and believe that I have an appropriate level of expertise to confirm that it is of an acceptable scientific standard.

# Peer review: Reviewer response for version 2

**DOI:** 10.5256/f1000research.126337.r134594

**Published:** 2022-04-25

**Authors:** Geoffroy Andrieux

**Affiliations:** 1 Institute of Medical Bioinformatics and Systems Medicine, Medical Center-University of Freiburg, Faculty of Medicine, University of Freiburg, Freiburg, Germany

Almansoori *et al*. presented a retrospective study on Thyroid cancer incidence in the United Arab Emirates. They collected data from GLOBOCAN repository and local study from UAE National Cancer registry. The authors have dissected the data from 2011 to 2017 and predicted the evolution up to 2040 using linear regression. The overall study is quite descriptive and informative. Nevertheless, the authors should address the following comments before indexing:

**Major comments:**

- Page 5 - "the data indicates that overall, the rate of increase of thyroid cancer cases is small compared to other cancers." Table 1 is not supporting this evidence since it only shows thyroid cancer cases. The authors should clarify this.
- Page 7 - The predicted and actual data correlate quite well (r = 92.5, p = 0.003). Is this correlation based on the total malignancies or thyroid cancer only? It would be nice to show both.
- Table 4 - The number of thyroid cancer cases among Emirati and expats should be either normalized to the ratio of Emirati and expats, or this percentages should also be investigated for other cancer types to see whether thyroid cancer has a peculiar distribution.
- Table 5 - The title mentions "nationality" but we do not see such distinction in the table.
- Table 5 seems to highlight male vs. Female difference and should rather be described after figure 3. Please double check.
- Figure 6 - It would be interesting to add another layer to see whether the female to male ratio is constant across the age groups or not.

**Minor comments:**

- Page 3 - There is a typo, "Global Caner Observatory" instead of Can **c**er.
- Figure 2 - the clustering is surprising since Europe seems to be the outlier and not Asia. I was wondering if the clustering was performed on the scaled data or not? Perhaps the authors can comment on this.
- Table 2 - Switch the first 2 columns to put the "actual" values first and then all the predicted values. Also "Thyroid cases" should be actually labelled as "Predicted Thyroid cases".
- Figures 4 and 5 - Please add a Y-axis description on the figure.
- Table 7 - A third column including the percentage of cases in each age group would be useful.
- Page 9 - "Table 7 and the cluster in Figure 6…", the word "cluster" might be misleading since there is no clustering applied on the heatmap, i.e. rows and columns are sorted by date and age respectively. I would suggest to rephrase this sentence.

Is the work clearly and accurately presented and does it cite the current literature?

Yes

If applicable, is the statistical analysis and its interpretation appropriate?

Yes

Are all the source data underlying the results available to ensure full reproducibility?

Yes

Is the study design appropriate and is the work technically sound?

Yes

Are the conclusions drawn adequately supported by the results?

Yes

Are sufficient details of methods and analysis provided to allow replication by others?

Partly

Reviewer Expertise:

bioinformatics, systems biology, systems medicine

I confirm that I have read this submission and believe that I have an appropriate level of expertise to confirm that it is of an acceptable scientific standard, however I have significant reservations, as outlined above.

# Peer review: Reviewer response for version 1

**DOI:** 10.5256/f1000research.80078.r128255

**Published:** 2022-04-05

**Authors:** Yuanping Zhou

**Affiliations:** 1 State Key Laboratory of Organ Failure Research, Guangdong Provincial Key Laboratory of Viral Hepatitis Research, Department of Infectious Diseases, Nanfang Hospital, Southern Medical University, Guangzhou, China

Almansoori *et al.* presented an interesting and useful article on thyroid cancer incidence in the United Arab Emirates. The article is concise and well written and the experimental part is well designed and some of the methods presented in the article can be applied to study the epidemiology of other rare tumours.

 There are two minor typos that needs correcting:

- Firstly, I think it is better for the title should be 'Thyroid Cancer incidence' (not the plural incidences).
- Secondly it will be more complete to include the equation for linear regression that the authors used, which I believe is y = m*x+c. This will clarify to the reader which of the equations was used in the study and allow for reproducibility of the results generated.

Is the work clearly and accurately presented and does it cite the current literature?

Yes

If applicable, is the statistical analysis and its interpretation appropriate?

Yes

Are all the source data underlying the results available to ensure full reproducibility?

Yes

Is the study design appropriate and is the work technically sound?

Yes

Are the conclusions drawn adequately supported by the results?

Yes

Are sufficient details of methods and analysis provided to allow replication by others?

Partly

Reviewer Expertise:

Clinical cancer and cancer epidemiology related investigation.

I confirm that I have read this submission and believe that I have an appropriate level of expertise to confirm that it is of an acceptable scientific standard.

# Response

**Published:** 2022-04-07

**Authors:** Rifat Hamoudi

**Affiliations:** University of Sharjah, United Arab Emirates

Dear Dr Zhou,

 Thank you for taking the time to review this article. We hope that the corrections below implemented in the revised will be to your satisfaction.

Comments

 Almansoori *et al.* presented an interesting and useful article on thyroid cancer incidence in the United Arab Emirates. The article is concise and well written and the experimental part is well designed and some of the methods presented in the article can be applied to study the epidemiology of other rare tumours.

 > We thank you for the constructive comments regarding the study.

 There are two minor typos that needs correcting:

- Firstly, I think it is better for the title should be 'Thyroid Cancer incidence' (not the plural incidences)

> In the revised version we have replaced the word "incidences" with the word "incidence"

- Secondly it will be more complete to include the equation for linear regression that the authors used, which I believe is y = m*x+c. This will clarify to the reader which of the equations was used in the study and allow for reproducibility of the results generated.

> We have added the line equation, y = mx + c to the methods section
